# Lake drying and livelihood dynamics in Lake Chad: Unravelling the mechanisms, contexts and responses

**DOI:** 10.1007/s13280-016-0805-6

**Published:** 2016-07-01

**Authors:** Uche T. Okpara, Lindsay C. Stringer, Andrew J. Dougill

**Affiliations:** Sustainability Research Institute, School of Earth and Environment, University of Leeds, Leeds, LS2 9JT UK

**Keywords:** Adaptive behaviours, Climate variability, Lake depletion, Livelihoods, Small Lake Chad

## Abstract

This article examines lake drying and livelihood dynamics in the context of multiple stressors through a case study of the “Small Lake Chad” in the Republic of Chad. Livelihoods research in regions experiencing persistent lake water fluctuations has largely focused on the well-being and security of lakeshore dwellers. Little is known about the mechanisms through which lake drying shapes livelihood drawbacks and opportunities, and whether locally evolved responses are enhancing livelihoods. Here we address these gaps using empirical, mixed-methods field research couched within the framework of livelihoods and human well-being contexts. The analysis demonstrates that limited opportunities outside agriculture, the influx of mixed ethnic migrants and the increasing spate of violence all enhance livelihood challenges. Livelihood opportunities centre on the renewal effects of seasonal flood pulses on lake waters and the learning opportunities triggered by past droughts. Although drying has spurred new adaptive behaviours predicated on seasonality, traditional predictive factors and the availability of assets, responses have remained largely reactive. The article points to where lake drying fits amongst changes in the wider socio-economic landscape in which people live, and suggests that awareness of the particularities of the mechanisms that connect lake drying to livelihoods can offer insights into the ways local people might be assisted by governments and development actors.

**Electronic supplementary material:**

The online version of this article (doi:10.1007/s13280-016-0805-6) contains supplementary material, which is available to authorized users.

## Introduction

Amongst the major global environmental changes observed around the world in recent decades is the drying of lakes (World Lakes Network 2004). Drying has taken place in, for example, the Aral Sea in Central Asia (UNEP 2014), Lake Chapala in Mexico (von Bertrab 2003), Lake Chilwa in Malawi (Njaya et al. 2011) and Lake Chad in west-central Africa (Odada et al. 2006). Numerous studies have stated that the diminution of lakes shapes the well-being and security of lake dwellers (Béné et al. 2003; Nindi 2007; Kafumbata et al. 2014). With some exceptions, empirical studies rarely examine the diverse range of mechanisms through which lake fluctuations influence livelihoods. Similarly, less is known about the range of opportunities lakeshore dwellers can access under lake-level fluctuations—including where lake drying fits in the suite of stressors affecting household livelihoods in water-limited environments that are increasingly a global focus of resource conflict concerns. As policy makers, managers and scientists work to address the changing climatic and socio-political landscape within which lake dwellers operate, there is a need for knowledge regarding the livelihood benefits that resource users lose or gain under conditions of water resource depletion, and in particular, how lake drying interacts with local contextual issues (White 2013).

Our goal in this article is to engage varied perspectives from individuals with livelihood patterns that combine subsistence agriculture with utilisation of lake water resources to better understand the underlying processes that set the context for livelihoods–environment links in regions facing persistent lake water fluctuations. Specifically, we seek to unpack how lake drying influences livelihood drawbacks and opportunities, as well as the range of mechanisms that shape the connections between these issues. To address these, we explore the dynamics of change in the livelihoods of lake dwellers, composed of farming, fishing and pastoral livelihoods, and the range of stressors confronting them. We equally assess where lake shrinkage fits amongst contextual stressors people experience and whether their portfolio of responses reflect locally adapted solutions. Our study is situated within the “Small Lake Chad” (SLC) basin, a region that is often presented as one of the most water-impoverished areas in the world (Singh et al. 2006) and has recently witnessed several sporadic outbreaks of violence, including terror attacks (Asah 2015). By studying the conditions and experiences of lake dwellers, the article points to a range of livelihood and lake-related concerns, and offers insights into the ways local people might be assisted by governments and development actors.

## Theoretical framework

Lakes hold more than 90% of the Earth’s freshwater resources (Rast 2014) and provide one or more of the supporting, provisioning, regulating and cultural services expounded within the ecosystem services framework (Millennium Ecosystem Assessment 2005). Temporal lake hydrological fluctuations undermine opportunities for agricultural livelihoods and exert significant effects on the well-being of human populations within and outside their physical basin (Nindi 2007). A livelihood is a means of gaining a living (Ellis 2000). Livelihood analysis considers how people and households combine and utilise different assets (human, natural, physical, social and financial) to pursue a livelihood strategy (e.g. farming) in order to generate a means of survival (Scoones 2009). Asset profiles are conditioned by past accumulation strategies and investments, which in turn are influenced by a range of contextual factors (social, economic, cultural, institutional and climatic) (Reed et al. 2013). Relationships between livelihoods and the environment can be understood through an assemblage of these contextual factors, either by disaggregated or aggregated research efforts (Jagger et al. 2012).

Household well-being is explicit in livelihood thinking and constitutes a positive livelihood outcome in terms of basic material for a good life, human security and better social relations (Millennium Ecosystem Assessment 2005). Rural people living in locations where their well-being is threatened often depend on a combination of income-generating activities, support from social networks and state-based safety net policies to develop adaptive capacities (Sallu et al. 2010). Abilities to respond to and withstand disturbances are often conceived as an attribute of robust livelihoods (Füssel and Klein 2006). Robust livelihoods display swift survival strategies; they also deliver better outcomes. Capacity to respond to perturbations, such as a shift in lake levels, may encompass reactive capacity (capacity to cope with and adjust to shocks or stresses/adverse conditions) and proactive capacity (capacity to search for and create livelihood options and strategies in order to increase competence with which to confront a threat) (Ifejika Speranza et al. 2014).

We envisage that reactive and proactive livelihoods will display different responses to lake water depletion and other contextual forces as they will use assets and construct livelihood strategies in different strategic manners (Paavola 2008). In settings with insufficient external assistance, multiple stressors can interact to widen the channels by which natural resource scarcity influences resource-dependent livelihoods (Tschakert 2007). In this study, we focus on the provisioning services of water supplies from the SLC in order to account for the diverse range of mechanisms by which lake water depletion can influence different livelihood groups and livelihood outcomes. Our emphasis on livelihood outcomes considers that if state or community-led water governance is predicated on a more complete understanding of how locals generate a means of survival, it can result in more effective lake resource conservation, promotion of livelihood security and reduced risk of local clashes.

## Lake Chad variability

**Fig. 1:**
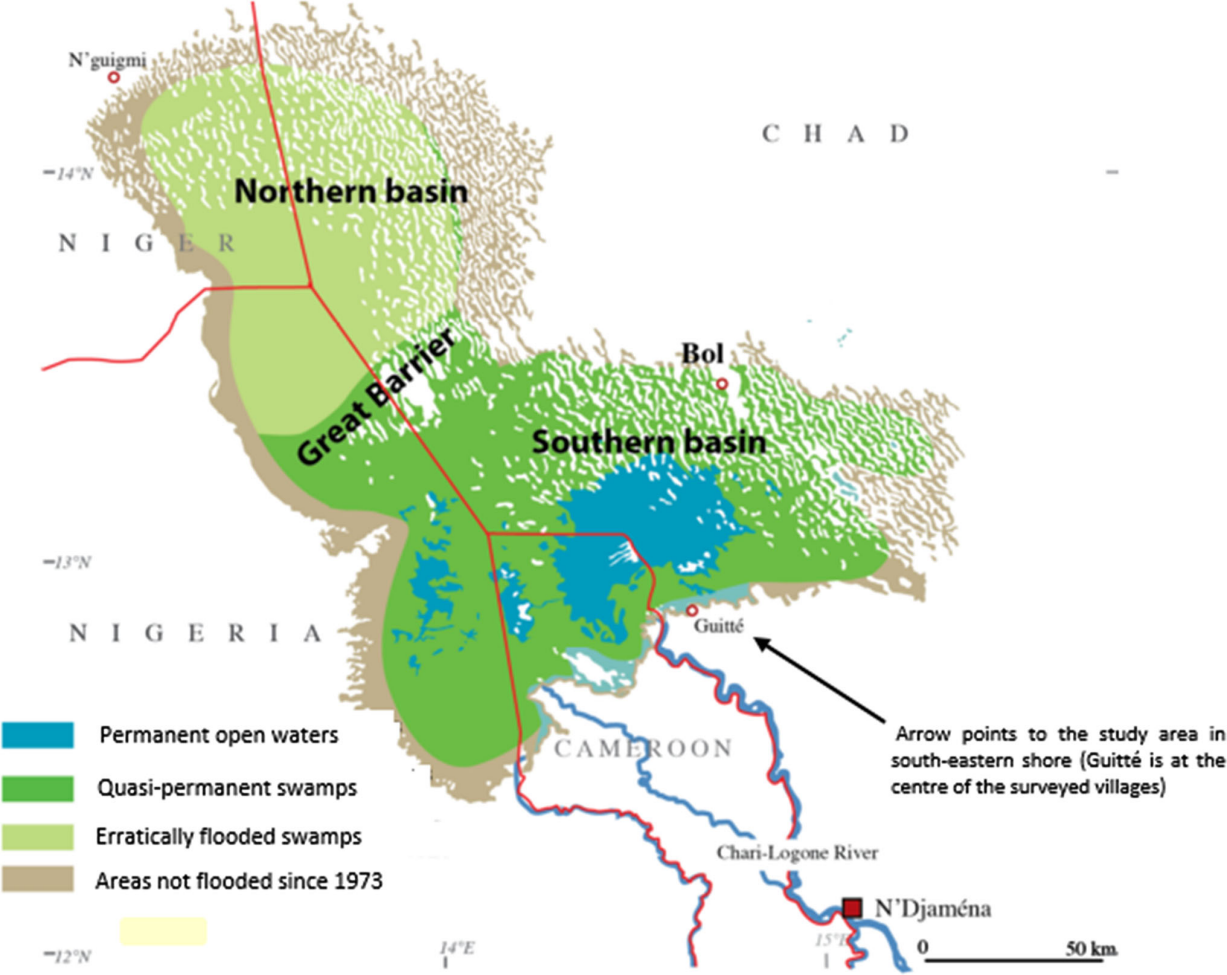
Map of the study area showing the average situation of Lake Chad in its ‘small state’ (2010–2015). *Source* Modified from Magrin et al. (2015)

**Fig. 2:**
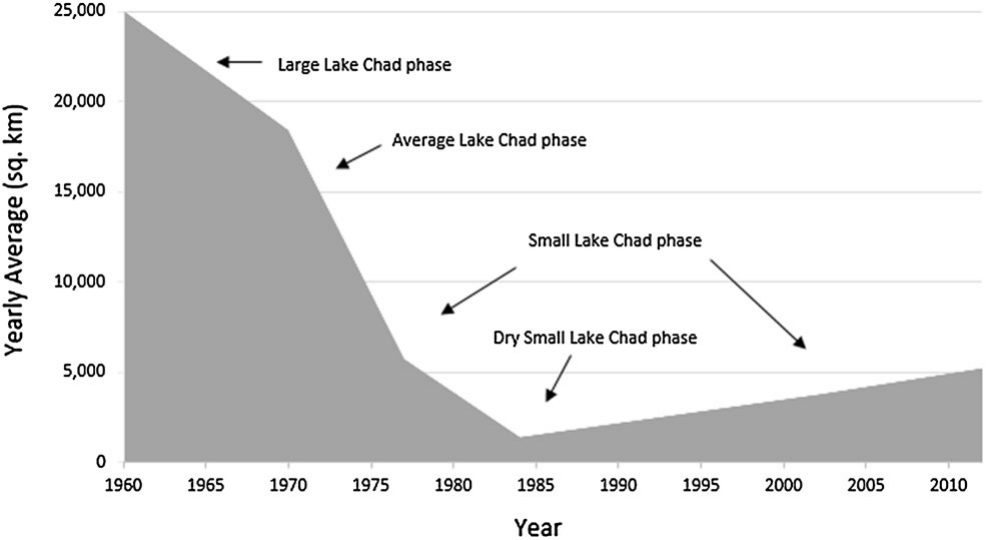
Open surface area of Lake Chad (stacked area graph with arrows pointing at lake levels for each specific phase—highlighting the various shrinking phases from 1960 to 2010). Data received from the lake Chad Basin Commission in N’Djamena

Lake Chad’s location, within the borders separating Chad, Cameroon, Niger and Nigeria, is geographically significant in west-central Africa because of its cultural and socio-economic prominence as the region’s agricultural heartland. At 25 000 km^2^ open water area in the 1960s, Lake Chad was the world’s sixth largest inland water body (LCBC 2014). The Lake today (Fig. 1) is a shrivelled, fragmented collection of two distinct water bodies, the northern and southern pools, dotting a drought-prone, desiccated landscape within the arid and semi-arid Sahel corridor. Satellite images and aerial photographs have been used to monitor the Lake’s fluctuations since the 20th century. Five major recession phases of the Lake occurred during the nineteenth and twentieth centuries, during which the Lake was alternatively described as a Mega, Large or Normal, Average, Small and Dry Small Lake Chad according to its water levels, depth and areal dimensions (Leblanc et al. 2011; Table 1; Fig. 2).

**Table 1:** The different phases/states of Lake Chad over time based on Lake Chad Development Expert Group Review, 2014 (cited in Mekonnen 2016)

Lake Chad phases					
Attributes	Dry small	Small	Average/medium	Large/normal	Mega
Inflows from the Chari-Logone (km^3^/year)	<15	15–34	35–43	>43	>50
Water level (m asl)	Dry northern basin	Different levels (<275)	280–282	>282.3	>285
Number of water bodies	Several	Several	One	One	One
Flooded area of the northern basin (km^2^)	0	0–8000	9000	10 000	>10 000
Dominant landscape	Swamps and savannas	Swamps/marshes	Dune archipelago	Open water	Wide open water
Aquatic vegetation	++	+++	++	+	+
Time period	Few years in the 1970s and mostly in the 1980s	1973 to present, except for “Dry Small” periods	1954–1972	1953–1954	Before the 1950s
Estimated size (km^2^)	500–1410	3000–14 000	18 000–22 000	22 000–25 000	340 000–400 000

**Fig. 3:**
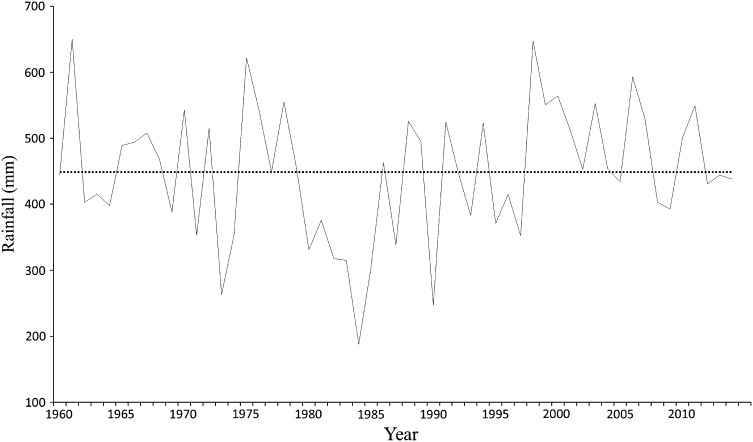
Rainfall trends for Lake Chad south-eastern shore. The figure illustrates total annual rainfall for the study area with *dotted line* representing the average values (449 mm) for the 1960–2014 period. It reveals substantial inter-annual rainfall variability typical of the Sahel belt. *Source* Statistics derived from climatological data obtained from the Directorate of Water Resources and Meteorology in N’Djamena

Causes of the shrinkage are reported by many (e.g. Gao et al. 2011; Lemoalle et al. 2012). They include increased irrigation withdrawals and decadal rainfall variability (Fig. 3), whilst the Lake bathymetry makes it vulnerable to water losses. The Lake region is increasingly challenged by population^1^ pressures (approximately 50 inhabitants per km^2^), water conflicts and changing agricultural practices. The lakeshore dwellers are poverty stricken—the location itself has a relatively high poverty rate (United Nations Human Development Report 2015). Other concerns include human illiteracy, health challenges exacerbated by the absence of good medical structures, and changing institutional policies on water use and fisheries management (Onuoha 2009).

## Methods

### Case study location and characteristics

Our study location is the south-eastern shore and islands of the SLC (Fig. 1) in the administrative department of Haraze Al Biar in the Chadian Hadjer-Lamis region (12°53″N; 14°37″E). Seven farming, fishing and pastoral villages (Table 2), were purposively selected based on their close proximity to the Lake, the dependence of the locals on the Lake waters, and the presence of specific climate markers (e.g. droughts and water scarcity). Existing SLC water bodies around the villages, including the permanent and seasonal ponds and the Lake’s open waters, provide important livelihood options in farming, fishing and livestock herding for the sedentary and nomadic population. Common crop types are maize, potatoes, soybeans, millet, wheat, sugarcane and vegetables (FEWS 2005). Soils close to the Lake are largely drought-prone, and can easily be flooded during extreme flood years (Luxereau et al. 2012). Property rights encompass *ndouba* (village owned) and private (landlord) arrangements, except for land within the lake floor, floodplain and wetland areas, which is held and allocated by the local authorities (the imams and bulamas) (USAID 2010). Fishing takes place both in the open water at the centre of the lake basin and along pools and water channels around villages and islands. Access to fishing, especially in open waters, is determined by the Chadian government and regulated by local authorities and the Joint Patrol Team (a team of regional security personnel guarding the open waters). Livestock herding is largely transhumant. The dominant ethnic group is the Arab shuwa. Other groups present include the Kotoko, Guran and Wadai. The common languages spoken are Shuwa/Chadian Arabic, French and Hausa.

**Table 2:** Characteristics of study villages and household surveys undertaken

Villages surveyed	Household size Mean^a^	Location^b^	Estimated^c^ households	Number of household heads sampled
Farming villages	8.68 (4.79)			
Miterine		Middle-distant	93	40
Guitte^d^		Near-to-road	186	80^d^
Fishing villages	8.71 (3.41)			
Kaesai		Remote island	70	30
Basara		Remote island	69	30
Kouri (Topio)		Remote island	47	20
Pastoral villages	8.80 (3.37)			
Dandi		Near-to-road/forests	70	30
Ngurutu		Remote camp	23	10
			(558)	(240)

^a^Values in parentheses are standard deviations

^b^Near-to-road village is within vehicle access (120–130 km) from N’Djamena and have a central market and bus/fuel stations; the middle-distant village is a long way off the paved roads (about 150 km from N’Djamena), accessible through unmarked tracks by motor bikes; remote islands where the fishermen live are accessible by boat or canoe; vehicle or motor bike access to remote locations is difficult without a guide who is familiar with the rough terrain. Access is usually not possible during rainy seasons

^c^Estimates are based on personal communications with local chiefs

^d^Guitte is a ‘mixed’ village where majority of households engage in either farming and herding activities or both; income-source ranking enabled the selection of 40 farmers and 40 herders from the village

### Data collection and analysis

Fieldwork was conducted in seven villages in mid-2013 and early 2014 using household surveys and semi-structured interviews stratified by farming (*n* = 80), fishing (*n* = 80) and pastoral (*n* = 80) livelihood groups; eight focus group discussions (FGDs) with different livelihood associations; and twenty key informant interviews (KIIs) with individuals with specialist knowledge on the study themes.^2^ We used three types of non-probabilistic sampling (cf. McCubbin et al. 2015). Purposive sampling selected a cross section of households^3^ in each of the villages (based on income contributions from their predominant livelihood activities). Non-proportional quota sampling ensured that an equal number of households within each livelihood group was recruited. Snowball sampling involved requesting the bulamas, the village research partners and the LCBC to recommend participants for the FGDs and KIIs.

**Fig. 4:**
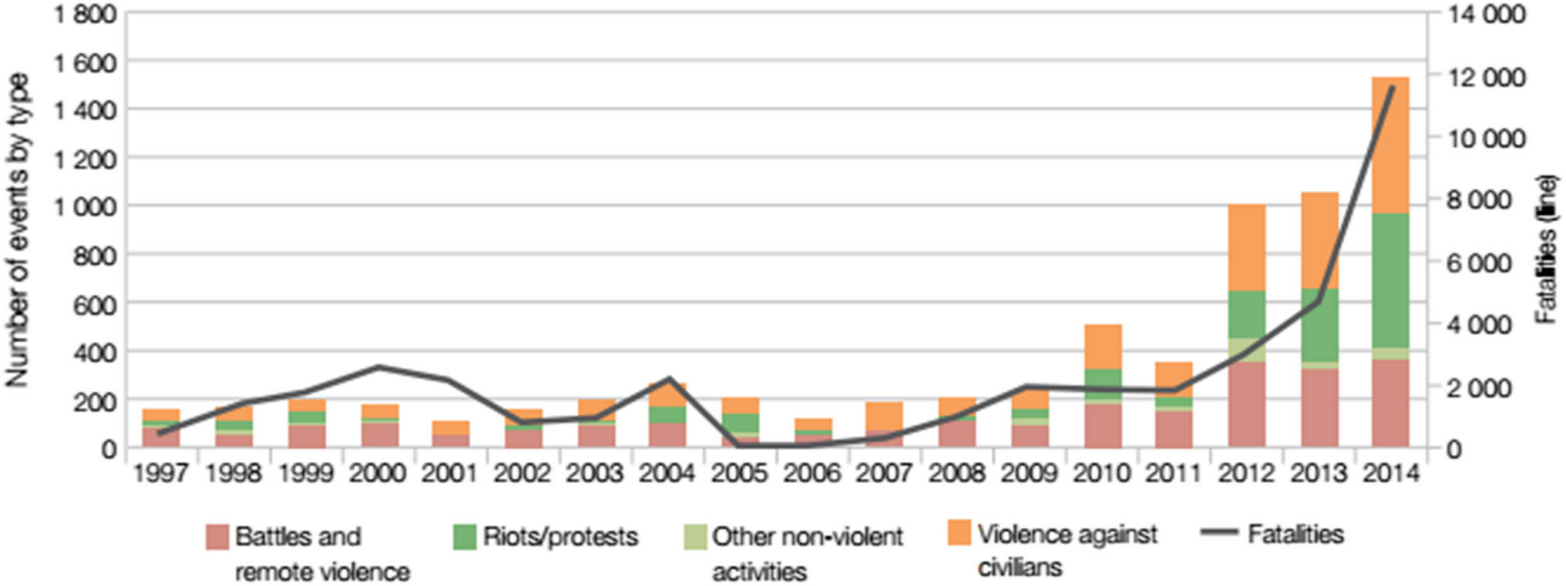
Trends of violence and fatalities in the Lake Chad region (1997–2014). *Source* Extracted from the Armed Conflict Location and Event Database (ACLED) version 5 (http://www.acleddata.com/data/version-5-data-1997-2014/)

Household surveys and interviews included questions categorised into themes based on the livelihoods framework (Ellis 2000). Data were collected on present household characteristics, assets owned (or can access), livelihood activities, conditions affecting the household, livelihood drawbacks and opportunities experienced due to the changing state of the SLC and other contextual factors, and the diverse portfolio of response strategies. The fieldwork drew insights from the reasons given for changes in livelihood conditions and strategies. It situated the role of the SLC in the context of other conditions, including climate variability (Fig. 3) and violent conflicts (Fig. 4), in order to tease out the major mechanisms through which the Lake has impacted livelihoods. Data collection was conducted in Arabic, French and Hausa, and translated at the time of collection. The socio-cultural and religious nature of the villages permits only males to grant interviews (see Béné et al. 2003), therefore only responses from male household heads were recorded.

Data from surveys, interviews and FGDs were supplemented with participant observation, village walks and secondary sources. Reflections on the data collected were undertaken jointly with key informants through continual dialogues/follow-up interviews. Data analysis was conducted at the household level and later aggregated at the livelihood group level. We assume the influences of the SLC to be homogenous within households, but heterogeneous across groups. Quantitative data were coded according to the broad categories in surveys for which statistical analyses were performed on the data in SPSS (v21). Qualitative data were coded in Microsoft Excel based on the broad themes from the survey and interview guides, after which qualitative thematic analysis was performed. We computed the average livelihoods diversification index across groups following Hahn et al. (2009) as the inverse of the number of livelihood activities + 1, reported by a household and aggregated across each group (higher values were assigned to groups with a lower number of livelihood activities). A stakeholder workshop convened in July 2015 at the Lake Chad Basin Commission headquarters, N’Djamena, enabled a robust triangulation and validation of our initial fieldwork findings.

## Results

### Unpacking the influences of the Small Lake Chad on rural livelihoods

#### The rural livelihoods contexts

Several households in the study villages are migrants (Table S1)^4^ from around the Lake basin. Their tendency to co-habit within small villages around one primary livelihood activity informed their placement into three livelihood groups. Table 3 sets out the assets held by different groups. More than half of all households, focus group discussants and key informants noted the relative weakness of household assets in terms of contribution to local livelihoods. The livelihood context is largely conditioned by high dependency on seasonal variations in natural assets. The SLC is a more accessible water source to farmers and fishermen than pastoralists, who on several occasions need to travel more than 50 km to the Lake’s water. Whilst fishermen who fish the open waters are required to pay certain charges, the wetlands and floodplains near several villages offer free fishing spaces for anyone during the rising flood seasons. The cultivation grounds of the Lake’s wetlands and recession beds provided fertile soils for food and cash crop cultivation in the past. Farming currently requires huge investments in fertilisers, pesticides and gasoline for motorised irrigation pumps to enable higher yields.^5^ Several farmers reported unimpeded access to arable land under the freehold and short-term use arrangements, except for the ‘new’ land spaces following the Lake’s contraction and in the forest areas. Landholding is based on smallholder arrangements over dispersed areas of 0.5–5 ha per household. Some fishermen and pastoralists who own or can access land sometimes engaged in crop cultivation as an additional activity.

**Table 3:** Descriptive statistics of asset variables for different livelihood groups derived from the survey data

Asset variable	Livelihood group^a^		
	Farmers (*n* = 80)	Fishermen (*n* = 80)	Pastoralists (*n* = 80)
Natural asset			
Water (for livelihood activities): households reporting SLC as the nearest water source for livelihood activity^b^	58 (72.5)	78 (97.5)	13 (16.3)
Land: household reporting unimpeded access to land	75 (93.8)	25 (31.3)	14 (17.5)
Financial asset			
Remittance: from members/relatives working elsewhere	18 (22.5)	36 (45.0)	30 (37.5)
Credit: households reporting access	24 (30.0)	8 (10.0)	15 (18.8)
Income: enough to cover important household expenses	29 (36.3)	19 (23.8)	6 (7.5)
Social asset			
Membership of social group	36 (45.0)	13 (16.3)	27 (33.8)
Access to institutions (e.g. LCBC) for external support	3 (3.8)	2 (2.5)	4 (5.0)
Group cooperation during challenges	44 (55.0)	53 (66.3)	23 (28.7)
Human asset			
Education: head started and/or completed primary school	13 (16.3)	20 (25.0)	21 (26.3)
Experience in livelihood activity (mean in years)	16.8 ± 12.7	14.2 ± 5.6	27 ± 8.1
Access to health care when sick	27 (33.8)	7 (8.8)	3 (3.8)
Household head (active age: 20–50 years)	61 (76.3)	65 (81.3)	66 (82.5)
Household depending majorly on one agro-based activity	64 (80.0)	49 (61.3)	64 (80.0)
Physical asset			
Housing: traditional (non-flood resistant) house	80 (100.0)	80 (100.0)	80(100.0)
Tools/equipment for livelihood activity^c^	78 (97.5)	75 (93.8)	12 (15.0)
Access to well, borehole, piped drinking water	72 (90.0)	8 (10.0)	23 (28.7)
Telecommunication: mobile phone	72 (90.0)	71 (88.8)	79 (98.8)
Average livelihoods diversification index	0.46	0.42	0.47

^a^Values in parentheses are percentages. Survey takes livelihood groups as the principal unit of analysis in line with the nature of the enquiry, therefore social differentiation within villages is not emphasised

^b^Local people at the southeast shore exploit various SLC (Small Lake Chad) water bodies, which include permanent and seasonal ponds, receding channels, Lake’s open water and water flows coming in from the Chari River

^c^Tools and equipment for livelihood activities are manual farming, fishing and herding tools

Almost all participants expressed concern over not receiving enough income to cover important household expenditures, such as food, water, housing and clothing. Access to remittances and credit is limited; a few households who have access reported that their incomes and livelihoods had not improved. Although membership in social groups that have productive livelihood benefits is low, several households attend mosques regularly, relate with religious doctrines that influence local cultural values and interact more readily during challenges (e.g. periods of low harvests, food and income) and festivals. These forms of social cohesion are common amongst farmers and fishermen who live a sedentary lifestyle. Although tendencies to specialise in one activity are high, a few households referred to having access to a range of different livelihood options that are not directly based on water and land, such as small trading in local markets, brick making, repairing of fishing nets and boats, and seasonal wage labour in nearby towns. Basic physical assets around villages and islands are dilapidated. Except for boreholes and mobile phone facilities that are accessible, schools, markets and hospitals are either non-existent or widely dispersed and poorly equipped. Poor rural roads affect the distribution of harvested produce. People live in weak, traditionally built houses, constructed with materials such as wood, sand, clay and papyrus, gathered around the Lake.

#### Influences of the Small Lake Chad

Interviewees, particularly village elders, recalled the ‘good’ times of the Normal and Average Lake Chad when rains were regular, and crop and fish yields were plentiful. During these phases, livelihoods benefited from a rich Lake ecosystem characterised by abundant flora and fauna. The Lake’s hydrological cycle enabled steady water supplies, and the region’s fishery productivity and food crop output flourished, whilst pastoralists had several pastures and succulent grasses (Odada et al. 2006). In contrast, the SLC phase is notable for the population pressure it triggered at the southern portion of the basin as the northern pool desiccated (Mekonnen 2016). This has caused new villages and temporary camps to emerge, whilst competition over limited resources intensified. The SLC phase first appeared in the mid-1970s during severe droughts in the Sahel region. It later re-appeared in the late 1990s and has since fluctuated between 3 and 14 000 km^2^. Although limited narratives prevent a complete understanding of livelihood conditions during the mid-1970s, pertinent to respondents’ current livelihoods is concern over local aggression towards the Lake’s changing state and declining income levels. Although aggressive attitudes towards land and water claims resulted in several conflict outcomes during the 1980s Dry Small Lake (Okpara et al. 2015), most respondents who reported aggression as a limiting factor stated it is often expressed through village-based urges to grab and scramble for scarce public resources, regular confrontations at water points, and limited sharing and cooperation.

Respondents could not accurately quantify their crop harvests, fish catches and milk/livestock sales over the past 5 years, but could identify trends in income status.^6^ Several farmers reported overall declines in crop outputs, although yields in some years (e.g. 2013) were better than others. One farmer in Guitte reported variations in the extent and timing of floods associated with the contraction of the SLC. These make it difficult for farmers to predict when in the year the floods would reach the farmland around their villages. This is particularly a concern to farmers who cultivate on the Lake beds as they regularly lose crops to floods. FGDs with farmers revealed a link between the current Lake state, water scarcity and low food production. Repeatedly, they referred to high costs of digging wells and pumping water (approximate costs range from USD110—180),^7^ and the labour involved in creating water channels into their farms. These limited their income from annual crop harvests.

Whilst a few fishermen stressed that fishing activities have not decreased, there was general agreement that the size and quantity of fish catches have declined. In 1 week, several artisanal fishermen would enclose approximately 120–180 small fish using hooks, a quantity that most fishermen of the full Lake era would catch in a single night. Fishermen generally complained about long distance fishing, high costs of renting or acquiring boats, strict fishing rules regarding the types of gear to use, high water access charges imposed by local authorities and the intrusion of unlicensed migrants from neighbouring countries, whose better fishing expertise often deny local fishermen access to the large fish. Similarly, pastoralists complained that their livestock were often sick and many had died with the decline in the richness and quality of the SLC pasture. Over half of the herders reported losing livestock in the last 5 years. Ten herders reported that milk output has declined from 2 to 5 litres to 1 to 2 litres per day. Key informants reported a series of other livelihood drawbacks linked to the SLC. Many agreed that the Lake’s shrinking limited local incomes and livelihood opportunities. Most referred to the Lake as creating a pool of unemployed people and provisioning convenient hideouts for insurgent activities, which had further increased the level of deprivation experienced by the locals in terms of physical, human and financial capitals. Increased outmigration, disease outbreak and low food quality were also noted (see Mekonnen 2016).

Current livelihood opportunities for lake dwellers centre on the renewal effects of the aquatic and soil environment associated with the seasonal floods, and the learning opportunities triggered by past droughts. Some farmers reported benefiting from flood-recession cultivation whilst several pastoralists utilised the Lake’s water for dry season herding. Inter- and intra-annual flood pulses enabled the recycling of the aquatic environment, providing multiple options for fishermen. Nearly 38 % of fishermen fished during previous floods and farmed on small land parcels after the floods. Key informants revealed that the flexibility of the Lake shore allowed for diversification and sustained regular interactions amongst migrants, but these often created inter-group competition. One key informant stated:

Two kinds of people exist around the SLC: those who take advantage of the dried Lake by seeking permission to cultivate the ‘new’ land areas and those who follow the Lake to new territories as it contracts. No matter the annual or seasonal condition of the Lake, people have always sought opportunities to better their lots (Key informant, July 2013).

Another said

The drying of the Lake has attracted several NGOs and institutions who often visit the areas for field surveys. What is happening now coupled with increased insurgency in the area has made the Lake a policy concern for riparian governments (Key informant, January 2014).

FGDs revealed that the ability of the lake dwellers to take advantage of existing opportunities depended on how long they have lived around the Lake and exploited the Lake’s resources, the range of social networks they can access and how proactive they can be during seasonal fluctuations. Many agreed that the lessons from the SLC period could spur new adaptive behaviours and learning, preparing the lake dwellers for possible future challenges, should sufficient external support become available.

#### Place-based stressors affecting livelihoods

Respondents noted social conflict, climatic changes and political/institutional instability as major livelihood stressors (see Table S2). Regular conflicts in the Lake Chad region have been reported resulting from environmental degradation, clashes amongst different ethnic groups and between locals and security officials at the Chadian shore (Fig. 4). The Lake’s geo-political location is characterised by instability which several key informants linked to past incidents of civil unrest in Chad, the arming of different ethnic militias and to recent terrorist threats. Periodic raiding of villages and the proliferation of military patrol checkpoints undermine livelihoods. Exertion of authority by the Joint Military Patrol at checkpoints often causes delays and adds unpredictable financial burdens to households moving their agricultural produce to markets. Challenges related to state regulations and local administration of rights to farmlands, pasturelands and open waters were reported during FGDs. High tax charges and inconsistent demands from local authorities constrain the asset profiles of lake dwellers, limiting their net income.

Regarding climate influences, especially rainfall shortages, one key informant at the office of the LCBC revealed that:

Current climate variability in the area is hard on the people and it is driving them into poverty; some commit crimes because it is increasingly becoming difficult for them to secure their livelihoods merely by farming or fishing or herding (July 2013).

Several livelihood groups that identified institutional instability (Table S2) related this to unjust governance over water and land whereby bulamas favour close associates and relatives when allocating resources. Although ethnic migrant influx (Table S1) has been increasing in the area, respondents complained that village elders/leaders are not often consulted before permits are granted to migrant fishers. This contributed to several conflicts experienced by locals on the Lake’s islands.

#### Local response strategies

**Fig. 5:**
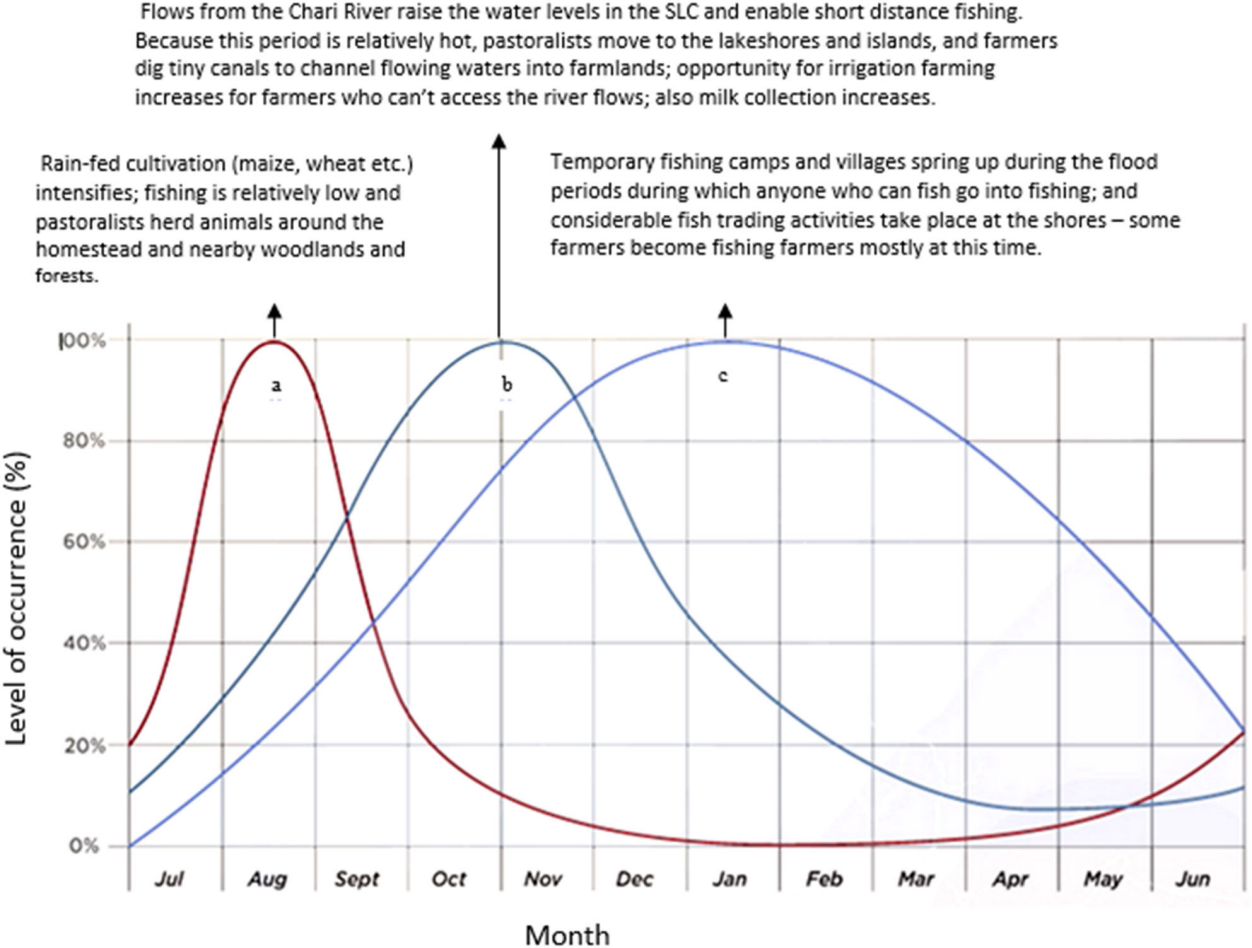
Annual flux of seasonal activities (based on Seasonal rainfall^a^, River flow^b^, Water level/flood^c^) observed during fieldwork at the south-eastern shore of Lake Chad, 2013–2014

**Fig. 6:**
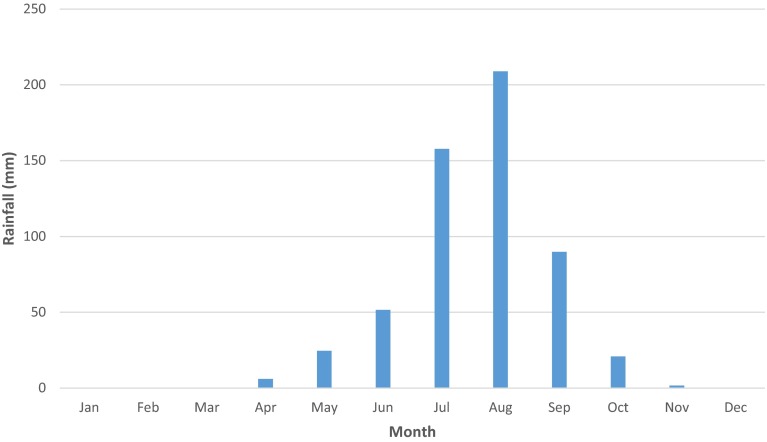
Mean monthly rainfall of the Lake’s southern shore near N’Djamena (1990–2014). *Source* Extracted from the World Bank Climate Change Knowledge Portal (2015) (http://sdwebx.worldbank.org/climateportal/index.cfm?page=country_historical_climate&ThisRegion=Africa&ThisCCode=TCD)

Livelihood groups employ a broad range of strategies to cope with and adapt to the conditions affecting their livelihoods. Table 4 summarises groups’ adaptive strategies and Fig. 5 shows the seasonal water conditions (in response to monthly rainfall patterns—Fig. 6), including the cycle of local activities. Farmers’ response strategies are largely agronomic and technological. More than half of the farmers followed seasonal patterns in their practice of mixed cropping, crop rotation, timing of land preparation, and planting and harvesting of crops. As indigenous water and soil ‘engineers’, they dig tiny canals through which water encircled their plots and remained as water reserves within the soil to curtail the effects of drought. This allowed crops to grow into maturity. They exploited water in wells or harvested ground water along different flooded water channels during dry months (November to March). Better-off respondents constructed farm-based dams or ponds to store water. Less than half have used improved crop varieties as they were not available locally. When land and labour are available and accessible, most farmers practiced intensification and/or extensification. They referred to cultivating three or more crops in the same or new units of land within the year, although cultivation was largely not mechanised. Farmers that traded or practiced short-term migration did so for economic reasons (e.g. to access land, credit, markets or new wage labour); and those engaged in tree crop cultivation and the exploitation of seasonal floods did so to take advantage of environmental opportunities for better livelihood outcomes. When farmers go fishing, particularly along small water channels during floods, it is usually for subsistence consumption. They often do not engage in pastoral activities, but keep small animals such as fowl, sheep and goats, which are sold to supplement household income.

**Table 4:** Contextual adaptive responses employed to deal with conditions affecting livelihoods. Compiled from household survey in the southeast shore of the Small Lake Chad

Adaptive responses^a^	Livelihood group		
	Farmers	Fishermen	Pastoralists
Agronomic-related	Changes in timing of land preparation, including planting and harvest dates Use of improved crop varieties when available (~45 % had access) Expansion of cultivated areas, including mixed cropping and rotation cropping (intensification and extensification as more land became accessible)		
Economic-related	~13 % trade in crops after peak farming activities are over	Migratory fishing in groups ~20 % practice recession cultivation and act in similar manner as farmers in terms of agronomic-related seasonal patterns of farming ~10 % trade in processed fish during low fishing periods	Collective livestock grazing and seasonal migration ~13 % cultivate crops when land is accessible
Environment-related	Afforestation: ~21 % plant trees usually around the homestead^b^ Exploitation of seasonal floods near the Lake is common	~12 % identified seasonal floods as suitable for increasing fish yield	~16 % often culled disease-infected animals ~8 % deliberately decrease herd size based on water and pasture availability
Technology-related	Ground water harvesting and storage of water in wells is common ~8 % construct small dams/ponds in farmland ~38 % can access weather information	~10 % cited using water from well occasionally for drinking Aspire better fish traps ~64 % use weather forecast	Well water is a backup against severe droughts; ~20 % harvest water along ground water channels ~33 % can access weather information

^a^The adaptive responses identified by respondents have been categorised into four here as agronomic (related largely to land and crop cultivations), economic (adjustments in behaviours to tackle the effects of low productivity), environmental (modifications in order to exploit the benefits of environmental changes either by tree planting, herd size reduction or exploiting seasonal floods), and technological (use of elements of local techniques in anticipation of future changes). A mix of strategies is common around the SLC

^b^Tree crops planted are mostly mangoes, banana, apple, cashew and orange trees—those planted on farmlands have their lower branches pruned regularly to enable minimal penetration of sun rays in order to help soils retain water and to prevent crops from drying out

Most fishermen fish in groups of two or more whilst exploiting the seasonal floods, a form of environmental advantage that allows for increase in fish yield. As various species (*Clarias gariepinus*—catfish, *Tilapiine cichlids*—tilapia, and *Alestes baremoze*—freshwater sardine) are caught, when the fish are tiny, fishers either created or accessed markets for tiny fish. As they live on islands and remote temporary camps close to the Lake, they fish within a short distance of their homes during the rising flood seasons, travelling far away when the floods recede. Most camps are evacuated during flood-recession periods, and fishers sometimes farm the ‘new’ lands within the camps. Almost all surveyed fishing households sold either smoked, dried, fried or fresh fish. Fishermen often desire better fishing tools, including safe and fast moving boats, but few can afford these. They can access weather information better than the two other livelihood groups largely because they are more concerned about water temperatures and wave/wind movements. Around the SLC, annual and long-term fish catch is difficult to estimate due to the large number of artisanal fishermen who do not keep records and the limited capacity of institutions and government agencies to collect and store fish catch data.

FGDs with pastoralists revealed that herders’ adaptive strategies address several challenges, including issues of sick animals and conflicts with farmers when animals stray into farmlands. Pastoralists maintain a specific herd size based on what they can manage or afford per annum, and also considering the availability of water and pasture around the SLC. Average cattle herd sizes are 53.65 ± 17.66 (ranging from 20 to 81 cattle). Camels, donkeys, horses, sheep and goats are herded too, but cattle remain the social identity and represent economic status amongst the nomads. As pastoralists’ income is largely from livestock-related activities, they utilise their social ties to identify favourable grasslands as they migrate seasonally in the surrounding SLC. Water storage in wells along several homesteads serves their water needs in dry seasons when the SLC is not close by. They camp around villages and forest woodlands, returning to the Lake areas during the dry season when they can ascertain that the grasslands and pastures are productive. Some respondents stated that when cows have enough grasses and water, they are able to produce 2–5 litres of milk per day which can add to household income.

## Discussion

### Mechanisms shaping the Lake’s influences on rural livelihoods

The Chadian shore holds a relatively large portion of the basin’s remaining open waters, and consequently has continued to create spaces for frequent trading and interactions amongst migrants of diverse ethnic groups (LCBC 2014). However, our findings show this advantage is yet to translate into better livelihoods for local people in the Hadjer-Lamis Lake Chad area. Farmers are burdened by low harvests despite that the SLC allows a combination of irrigation, flood-recession and rain-fed cultivation. Opportunities for seasonal mobility exploited by pastoralists have often pitched them against other resource users as aggression over limited supplies of water and grasslands intensifies. Fishermen exploit various water bodies seasonally, yet increased fishing activities have not led to increased catches. FAO (2012) reported a decrease from 220 000 tonnes of fish in the 1960s to about 100 000 tonnes in 2000. Recent annual yields are placed at 50 000 to 60 000 tonnes (Murray 2007). Similarly, low outputs have been reported for crops (e.g. sorghum declined from 328 000 tonnes to about 180 000 tonnes between the late 1960s and the years following 2010) and livestock—declining at nearly 2 % per year since the 1960s (Ovie and Emma 2012; Mekonnen 2016).

There is strong evidence that when households lack important livelihood assets, especially human, financial and social capitals, changes in environmental variables (affecting natural capital) shape the processes of household decision making, constraining livelihood options (de Sherbinin et al. 2008; Musoke and Boon 2012). Locals have diverse socio-economics needs which existing asset portfolios, including the changing Lake water system, cannot satisfy. Several livelihood groups were unable to secure enough income to cover basic materials for a good life. Although our findings indicate that the provisioning services of the SLC waters created a range of livelihoods drawbacks and opportunities, the major mechanisms that shaped these stem from the: (1) way different livelihood groups are inflexibly tied to water-dependent activities, (2) tendency to focus on one primary livelihood activity due to limited opportunities outside agriculture (restricting diversification and asset accumulation), (3) influx of the mixed ethnic migrants, (4) manner in which the Lake provides spaces for different terrorist activities and violent conflicts, and (5) limited village infrastructure.

The shrinking of the SLC rarely affects livelihood groups directly. Asset holdings derived from unstable water-based activities are a medium through which water depletion influences livelihoods. The tendency to specialise in one primary activity for survival made such activity the delivery agent of many of the impacts of SLC water-level fluctuations for the different livelihood groups. This resonates with observations by Kafumbata et al. (2014) for Lakes Chilwa and Naivasha in Malawi and Kenya, respectively. Aggression and contestation amongst the mix of migrant ethnic groups, including the role seasonality plays in shaping social interactions and the scramble for scarce resources, offer explanatory power in understanding the influences of the SLC on livelihood groups. The evidence presented demonstrates how sources of livelihood drawbacks can become more obvious in a setting where lake depletion opens up remote spaces for inter-group clashes and other activities that undermine human security (see Taguem Fah 2007; Ifabiyi 2013). Scarce local infrastructure, for example, local markets, weaken trade networks and prevent people from earning sufficient income from the sale of agricultural produce. Lack of amenities, as reported by several participants and evident in much of the Hadjer-lamis region, presents another medium through which the influence of the Lake’s depletion is experienced. Sarch (2001) has argued that governments often display unwillingness to invest in infrastructural development at lakeshore locations whose future is uncertain or whose jurisdiction is unclear. This is particularly the case on islands where local people use the Nigerian currency, yet they are located within the Chadian shores where the Central African CFA franc is the common medium of exchange.

### Where does the Small Lake Chad fit amongst other stressors?

Resource poor locations are constrained by multiple stressors (Tschakert 2007). Climate, conflict and governance issues, identified by the participants as key interacting stressors affecting people’s livelihoods, were long a concern in the Lake region (pers. comm with a security guard, Guitte, July 2013). To demonstrate where the SLC fits amongst these stressors, we used a disaggregative approach (cf. Leauthaud et al. 2013). This showed that the SLC has become interwoven and deeply rooted in the fabric of local livelihood contexts that influence the assets of resource-dependent households. Analysis of the Lake’s influence points to its contributory role in driving vulnerability to climate and non-climatic conditions through the water channel (Ovie and Emma 2012). In particular, it has been argued that the underlying conditions fuelling social and political instability, including climate variability are shaped by the SLC (Onuoha 2009; Lemoalle et al. 2012). For example, the Lake’s depletion constrains its ability to serve as a hydrological buffer against drought events in much of the dryland area. Weakened social ties due to struggles over limited water supplies, including limited income opportunities caused by unfavourable tax regimes on the open water fishing activities, point to some of the ways the SLC fits amongst the stressors influencing the development trajectories of lakeshore villages and islands.

Observations that water depletion represents a major channel through which much of the SLC roles fit into the context of other stresses are consistent with: Kreamer’s (2012) description of water shortages as a facilitator of tribal violence and cross-border terrorism; Ludwig et al.’s (2011) illustration of water scarcity in climate-conflict interactions in settings where socio-cultural and political structures are weak; and Tir and Stinnett’s (2012) argument for water governance challenges in situations where a water resource traverses national boundaries.

### Are locally evolved responses suggestive of locally adapted solutions?

A range of local responses to survive the challenges posed by the changing Lake waters were identified. Locally evolved responses indicate how decisions based on seasonality and traditional predictive factors are predicated on the availability of capital assets. For example, better-off farmers who constructed small dams and ponds to store water were able to cultivate more crops on bigger land areas and remained productive across the seasons. This type of proactive response keeps livelihoods functioning, and enhances resilience against adverse water conditions (Ifejika Speranza et al. 2014). Because many adaptive options to environmental changes depend on using, combining or substituting assets in different ways (Reed et al. 2013), the low asset profiles of several households limited them to responses that are largely reactive and as a result, constrained capacities to create locally adapted solutions that both reduce poverty and enhance well-being. Basic materials were in short supply due to the decrease in water available for soil fertility and plant growth, including fishery and livestock productivity. This led to a sense of deprivation within local populations, especially as access to external support remains limited. With the added burden of fear of militant Islamists (Taguem Fah 2007) and the loosening of social cohesion, opportunities through which groups and households could enhance response strategies were blocked.

How seasonal patterns and water conditions might change in the future for locations in the Sahel region is unclear (Cook 2008). If local people experience difficulty in predicting the timing and cycle of hydrological changes, deciding when and how to adjust livelihood activities might become challenging. This could further undermine local livelihoods, reducing adaptive options. Negative changes in the wider social (e.g. conflict), economic (e.g. income levels) and political/institutional contexts will create additional burdens in ways that could broaden the mechanisms through which lake water depletion influences agricultural livelihoods.

## Conclusion

This article uses primary information accessed by studying the conditions and experiences of lake dwellers to pin down the mechanisms shaping how lake drying influences livelihoods, including the range of stressors confronting people that are tied to water-based activities, and to identify how seasonality spurs reactive behaviours. Findings underline how a relatively homogenous livelihood structure, low asset profiles, limited village infrastructure and a conflict-prone environment influence livelihood drawbacks and opportunities. The findings point to several livelihood concerns requiring development assistance. Of particular importance is assistance that targets the low infrastructure profile of the lake region, including agricultural production practices anchoring rural livelihoods. Such assistance would also need to target the socio-economic, political and human insecurity factors shaping influx of migrants. The recent joint inter-governmental efforts to restore human security in the Lake Chad basin offers some hope, optimism that the Lake Chad Basin Commission (LCBC 2014) has demonstrated in a strategy to revitalise the lake waters and to rebuild the resilience of local resource users. Aside from being of academic interest to development scholars and others engaged within the human-society-environment research arena, the findings presented in this paper have resonance for the lake dwellers. For these locals, the condition of the Small Lake Chad represents much more than a photograph of a drying, fragmented collection of water bodies. Lake drying has meant a destruction of the basic materials needed for a good life, as well as an unwelcomed assault on social security and economic livelihoods. As physical insecurity is carried into different riparian territories (Asah 2015), as fears of insurgency increase (Taguem Fah 2007), and as lake drying and low human asset profiles fan social unrest (Onuoha 2009), the findings presented here have global significance as well. This study, if nothing else, calls for a reconsideration of the livelihood challenges inherent in the drying Lake Chad region. The impassionate call is unmistaken—the Small Lake Chad region’s ecological and livelihoods resilience is at stake and there is an urgent need for interventions that can enhance and enable scope for asset accumulation, co-habitation of mixed migrants and easy access to opportunities outside lake-based activities.

## Electronic supplementary material

Below is the link to the electronic supplementary material.

Supplementary material 1 (DOC 43 kb)

## Footnotes

1 Annual population growth rate is estimated to be 2.5–3.0% (UN Population Division 2015). Inhabitants of the Lake have access to less than 550 m^3^ of water per year—they increasingly experience a condition of absolute water scarcity (Mekonnen 2016).

2 These are officials of the Lake Chad Basin Commission (LCBC), government ministries, lakeshore patrol guards and other external stakeholders.

3 We estimated household populations per village (Table 2) based on personal communications with local chiefs and applied a quota scheme in which 43% of estimated households per village were selected—this constituted a manageable unit for closer investigation considering the transient lifestyle of majority of the respondents.

4 See Table S1—Several are either internal or cross-border migrants settling temporary or permanently, who move due to economic and environmental constraints, including conflict threats. They are spurred by the need for survival; and because the attractiveness of the lake resources heightens during dry periods, migrants travel from far away islands and hinterlands, as far as several hundreds of kilometres to the lake water areas (see Okpara et al. 2015).

5 This emerged from interview conversations with local experts and agrees with evidence in previous studies (e.g. Luxereau et al. 2012; Ovie and Emma 2012).

6 In Chad generally, Multidimensional Poverty Index is estimated to be 0.545; intensity of deprivation is put at 63 % with approximately 87 % of households living in poverty (the 2005–2015 survey results on poverty for this region are available in the UN Human Development Report of 2015).

7 Locally, this is approximately CFA france 106 070; a cost that is very high for majority of the local resource users.
