# Supplementary material for: Lake drying and livelihood dynamics in Lake Chad: Unravelling the mechanisms, contexts and responses
Source: Ambio. 2016 Jul 1;45(7):781–95. doi: 10.1007/s13280-016-0805-6 (PMC5055484; doi:10.1007/s13280-016-0805-6)
Supplement: Supplementary file 1 — Supplementary material 1 (DOC 43 kb) [file 13280_2016_805_MOESM1_ESM.doc]

Electronic Supplementary Material

Title: **Lake drying and livelihood dynamics in Lake Chad: unravelling the mechanisms, contexts and responses**

Authors: Uche T. OKPARA, Lindsay C. STRINGER, Andrew J. DOUGILL

Table S1 Migrants in the sample: mean length of residency/mobility based on the 2014 survey exercise

| Livelihood group | Migrants in sample | Mean length of stay/mobility (years) ^a^ | Ethnic affiliation (dominant groups) ^b^ |
| --- | --- | --- | --- |
| Farmers | 55 (69%) | 18.29 | *Arab* (29%),  *Wadai* (20%),  *Guran* (13%) |
| Fishermen | 62 (78%) | 16.42 | *Kotoko* (30%),  *Guran* (20%),  *Masai* (16%) |
| Pastoralists ^c^ | 64 (80%) | 22.75 | *Arab* (48%),  *Guran* (34%),  *Fulani* (19%) |

^a^ Length of stay/residency implies the number of years migrant resource users have lived in or traversed the Lake Chad area where the survey was undertaken.

^b^ Mixed ethnic groups characterise the southern Lake Chad area – the dominant groups are shown in the table. The human population dynamics around Lake Chad are often driven by a southward migration trend (spurred by the need for survival) since the collapse of the lake’s northern pool in the 1970s and 1980s (Okpara *et al*. 2015).

^c^ Pastoralists are very mobile groups; for them the lake represents a shelter during exceptionally drought periods and thus serves as the heart of a mobile radius/system across seasons.

Table S2 Identified stressors affecting livelihoods in the Small Lake Chad area

| Stressor (% of respondents: ‘aggregated’) | Description of stress condition based on FGDs, KIIs and previous studies | Sample quote (s) |
| --- | --- | --- |
| **Conflict**  Conflict in the SLC (89%)  Losses/injury from conflict  (44%)  Feeling insecure (45%))  Participation in conflict (54%) | ....the succession of civil wars in Chad, the arming of militias from different ethnic groups, and recent terrorist threats create insecurity, thus villages are continuously raided and livelihood resources destroyed ^a, b, d^ | “People are now used to not having the lake around, so everyone just goes about as if the lake doesn’t exist, thereby creating space for regular contacts and conflicts between locals and foreigners”. (Fisherman respondent in Basara, March 2014)  “Before people were selfless, less suspicious and liberal with their possessions. That has changed now. Deprivation is increasing and struggles for survival keeps driving people into conflict behaviours, making this area very insecure for humankind” (Farmer respondent in Miterine, July 2013)  “Psychologically, people's mind-sets are changing as the environment is changing; people are more careful now with their relationships with others, lack of understanding causes in-fighting here…on the surface people move about as if nothing is happening, but there are underlying issues of discord that are unaddressed”. (Pastoralist respondent in Ngurutu, July 2013) |
| **Climate**  Long-term climate shifts  (>10 years) – (100%)  Climate impact-related losses  (96%) | ....increasing droughts (reduced rainfall) and uncertainties regarding the timing and extend of inter- and intra-annual floods pose a threat to livelihoods ^a, b, c^ | “There’s a lot of complain about bad harvest here; what you put in the soil often turns out bad in the end due to hot temperatures, and this makes commercial farming difficult”. (Farmer respondents in Guitte, February 2014)  “I have noticed we no longer receive our seasonal rainfall in its time. Our dry season has extended, shortening our rainy season. Dry seasons make ‘big catches’ difficult…we are further impoverished as a result”. (Fisherman respondent in Kaesai, February 2014) |
| **Political/institutional instability** (85%) | ....organisations charged with regulation of resources are not well funded….officials extort money from locals indiscriminately ^a^  ...regional instability negatively influences government willingness to invest scarce resources in infrastructural development at village locations whose future is described as uncertain ^a, d^ | “Life is hard, people and communities lack so many basic amenities and our government does not seem to care”. (Pastoralist respondent in Dandi, March 2014)  “Four national governments hold the lake waters, their agents come here regularly to ask us questions. We supply them relevant information and expect their assistance. Yet illegal fishing has continued and the security men often interrupt our fishing activities” (Fisherman respondent in Kouri, July 2013) |

^a^ Responses from key informant interviews (March 2014)

^b^ Response from FGDs (March 2014)

Other studies that identified similar stressors around the wider Lake Chad area:

^c^ Onuoha (2009)

^d^ Sarch (2001)

(Note: readers are referred to the reference list in the main article for the cited sources mentioned here)
